# P-93. Contezolid in Patients with Spinal Infections

**DOI:** 10.1093/ofid/ofae631.300

**Published:** 2025-01-29

**Authors:** Bo Wei, Xiucui Zhang, Jianrong Wang

**Affiliations:** Changzheng Hospital, Second Military Medical University, Shanghai, Shanghai, China (People's Republic); Changzheng Hospital, Second Military Medical University, Shanghai, Shanghai, China (People's Republic); Changzheng Hospital, Second Military Medical University, Shanghai, Shanghai, China (People's Republic)

## Abstract

**Background:**

Methicillin-resistant *Staphylococcus aureus* (MRSA) is a common cause of spinal infections (SI) requiring long-term use of antibiotic therapy. Linezolid (LZD) is effective in treating MRSA in different clinical scenarios, but its long-term use is limited due to risk of significant thrombocytopenia. Contezolid (CZD) is a new generation of oral oxazolidinone with potentially less myelosuppressive effect. However, data on the use of CZD in patient with SI is currently lacking. This study aims to evaluate the use of oral CZD in patients with spinal infections due to MRSA.

**Figure:**
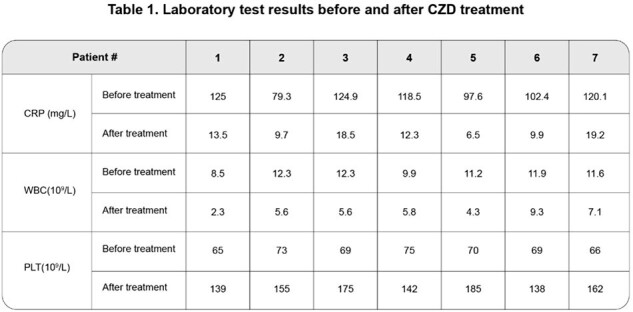

**Methods:**

Single-center, retrospective study in 7 patients with SI caused by MRSA. All patients experienced platelet count decreased during treatment with LZD and were rapidly switched to CZD (800 mg, po, bid) for > 4 weeks. We evaluated the clinical outcomes (within six months after treatment), adverse reactions and the platelet counts.

**Figure:**
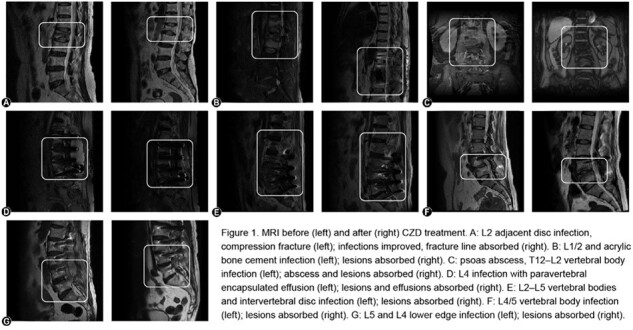

**Results:**

7 patients met the criteria for this study (median age 58, IQR 56.5-64.5, 14.3% female). With LZD treatment for a median of 14 days ( IQR 12-14 ), platelet count decreased from normal range 125-300 × 10^9^/L to 65–75 × 10^9^/L. After switching to CZD for 2 weeks, the platelet counts recovered to > 100 × 10^9^/L in all subjects. At the time of the switch 5 patients still had increased C-reactive protein. After approximately 2 months of oral CZD treatment(median duration 28 days, IQR 26-31.5 ), all patients improved clinically and CRP levels returned to normal (Table 1). Follow up MRI results also indicated radiologic improvement (Figure 1). Clinical evaluation, CRP and MRI six months after therapy indicated no recurrence in any subjects. During the treatment with CZD, only 2 patients experienced mild nausea, with no serious adverse reported.

**Conclusion:**

In patients with SI due to MRSA experiencing thrombocytopenia with LZD, This limited study of oral CZD, as a rescue therapy for hematogenous osteomyelitis and thrombocytopenia after treatment with LZD, is encouraging and suggests that CZD may with more study be an excellent option for the treatment of SI due to MRSA.

**Disclosures:**

**All Authors**: No reported disclosures

